# Prediction of BOS by the single-breath nitrogen test in double lung transplant recipients

**DOI:** 10.1186/1756-0500-4-515

**Published:** 2011-11-26

**Authors:** Gerdt C Riise, Gunnar Mårtensson, Birgitta Houltz, Björn Bake

**Affiliations:** 1 Department of Respiratory Medicine and Allergology, Institute of Medicine, Sahlgrenska Academy, University of Gothenburg, Gothenburg, Sweden; 2 Department of Clinical Physiology, Institute of Medicine, Sahlgrenska Academy, University of Gothenburg, Gothenburg, Sweden

## Abstract

**Background:**

The present study analyses the ability of the alveolar slope of the single-breath nitrogen washout test (N_2_-slope) to diagnose and predict the development of the bronchiolitis obliterans syndrome (BOS).

**Methods:**

We present a retrospective analysis of 61 consecutive bilateral lung or heart-lung transplant recipients who were followed at regular control visits during a three year follow-up. The operating characteristics of the N_2_-slope to diagnose BOS and potential BOS (BOS 0-p) and to predict BOS were determined based on cut off values of 95% specificity.

**Results:**

The sensitivity of the N_2_-slope to identify BOS was 96%, and BOS 0-p 100%. The predictive ability to predict BOS with a N_2_-slope > 478% of the predicted normal was 56%, and if combined with a coincident FEV_1 _< 90% of the basal value, the predictive ability was 75%.

**Conclusions:**

The predictive ability of either the N_2_-slope or of FEV_1 _to diagnose BOS is limited but the combination of the two appears useful. Follow-up protocols of bilateral lung and heart-lung transplant recipients should consider including tests sensitive to obstruction of the peripheral airways.

## Background

Bronchiolitis obliterans is the leading cause limiting long-term survival after lung transplantation [1]. The prevalence is 50 to 60% in long-term survivors and account for 30% of all deaths after the third postoperative year [2]. The pathogenesis and pathophysiology are not fully known and the effect of treatment regimens has been disappointing. During recent years new treatments have been attempted but the effect on long term survival has so far been limited [3,4]. It is likely that an early diagnosis is potentially favourable by leading to earlier initiation of therapy and possibly an improved prognosis.

A histologic diagnosis of bronchiolitis obliterans is usually not obtained due to the insensitivity of transbronchial biopsies [5]. The term bronchiolitis obliterans syndrome (BOS) was therefore introduced and based on a ≥ 20% decrease in FEV_1 _rather than on histology [6]. BOS stage 0-p was put forward by Estenne et al in an update of the original staging system in order to identify potential BOS patients and was defined by a decrease in FEV_1 _between 10% to 19% from the baseline value [7]. The stage BOS 0-p, based on the FEV_1 _criterion, has since been reported to be "a reasonable predictor of BOS stage 1 after bilateral lung transplantation" [8].

In a preliminary report in 1997 [9], we showed that uneven intrapulmonary ventilation distribution, as indicated by an increased alveolar slope of the single-breath nitrogen test (N_2_-slope) [10], seemed to precede the decline of FEV_1 _in BOS patients. This finding has since been confirmed by Reynaud-Gaubert et al [11] who reported a high sensitivity but low specificity of the N_2_-slope to diagnose BOS. Estenne et al [12] measured the alveolar nitrogen and helium slopes following inhalation of one litre of a gas mixture containing helium and reported that raised slopes preceded the development of BOS, but the associated specificities were rather low. The sensitivity of a test is defined by the chosen threshold value and a high sensitivity may be obtained on the expense of a low specificity. Therefore, these sensitivities are difficult to compare and interpret.

The ability of a lung function test to diagnose a disease is usually based on its lower limit of normal, generally defined as the 5^th ^or 95^th ^percentile of values in a normal population. Regarding double lung transplant recipients, "normal values" are almost certainly different from a healthy population, and the lower limit normal should preferably be defined by the 5^th ^or 95^th ^percentile of transplanted patients without known graft complications.

The aims of the present analysis was first to analyse the operating characteristics of the N_2_-slope to diagnose BOS and BOS 0-p by applying a cut off value chosen to give a specificity of 95%, and secondly to analyse the ability of the N_2_-slope to predict the development of BOS.

## Methods

A total of 92 patients underwent heart-lung or bilateral lung transplantation between January 1990 and April 2003 in the Gothenburg Lung Transplantation Program. The study design was approved by the Ethical Committe of the University of Gothenburg (Dnr. 044-05).

The present study is a retrospective analysis of the 61 patients available at a two year follow-up examination. 31 patients were not included (21 had died, 9 were incapacitated due to post-transplant co-morbidity and 1 patient was unable to reach). Fifty-two patients took part in a three year follow-up examination. Out of the included 61 patients, 24 patients were heart-lung transplanted and 37 patients had undergone bilateral lung transplantation, 33 were women and 28 men. Their mean age was 34 years (range 7 - 58). The preoperative diagnoses were primary pulmonary arterial hypertension (n = 17), Eisenmengers syndrome (n = 17), cystic fibrosis (n = 11), chronic obstructive pulmonary disease or alfa-1-antitrypsin deficiency (n = 4), idiopathic pulmonary fibrosis (n = 2), and various other diagnoses (n = 10).

During the follow up period, all patients received a standard immunosuppressive protocol of cyclosporine, azathioprine and steroids. Regular visits at the transplant unit including clinical, radiological and pulmonary function examinations, bronchoscopy with bronchoalveolar lavage and transbronchial biopsies took place 1, 2, 3, 4.5, 6, 9, 12, 18, 24 and 36 months post transplantation. A detailed account of the follow-up regimen has earlier been published [13].

Pulmonary function tests were performed by experienced and skilled technicians in a specialized respiratory laboratory. The quality control and performance of spirometry, measurements of lung volumes and carbon monoxide uptake (CO-uptake), *i.e*. transfer factor or diffusing capacity, were in accordance with the European recommendations [14,15]. Spirometry was performed on a rolling seal spirometer (SensorMedics 922, SensorMedics Co., Yorba Linda, CA, USA). Volume calibration was checked daily. In almost all instances three acceptable recordings were obtained. The forced expiratory vital capacity (FVC) and FEV_1 _were taken as the largest from the acceptable recordings. Lung volumes, i.e. total lung capacity (TLC), functional residual capacity (FRC) and residual volume (RV) were obtained in a body plethysmograph (SensorMedics 2800, SensorMedics Co., Yorba Linda, CA, USA). Volume and pressure calibrations were checked before each measurement and the panting frequency was kept below 1 Hz. CO-uptake was obtained by the single-breath method using standard equipment (SensorMedics 2200, Sensor Medics Co., Yorba Linda, CA, USA). Volume and gas concentration calibrations were checked daily.

The N_2_-slope was determined by a homemade bag in box system [16] using a rapid nitrogen analyzer (Med. Science Nitralazer 505). The N_2_-slope was determined by a defined algorithm [16,17] with minimal possibilities for subjective influence. Volume and nitrogen calibrations were checked at each investigation and the tracings were read by a laboratory physician without knowledge of the status of the patient. Two acceptable recordings were obtained in almost all instances and the average value used for further analysis.

Predicted normal values for spirometry and lung volumes are according to Quanjer et al [15], CO-uptake according to Salorinne [18], and the N_2_-slope according to Sixt et al [19].

BOS and BOS 0-p were diagnosed according to the 2001 update of the diagnostic criteria [7] but without considering FEF_25-75 _as it was not measured at the time. Baseline values were calculated for all variables and based on test results as from 4.5 months after transplantation. Test results obtained at the various instances were then expressed as a percentage of the latest baseline value, similarly as for FEV_1_. Calculations of cut off values were based on all test results between 6 and 36 months of the 43 stable patients and were chosen to result in 95% specificity.

The ability of the N_2_-slope to predict BOS was determined by the ratio of abnormal results (i.e. outside the cut off value) preceding the diagnosis of BOS and the sum of all abnormal test results among patients without BOS. Expressing this ratio as a percentage signifies the probability of an abnormal result to be associated with the development of BOS within the follow up period. Results as from 6 months post transplantation were considered in the analysis. Three patients were excluded in this analysis and in the analysis of operation characteristics as they were diagnosed with BOS 0-p at the final visit and consequently the relation to BOS is unclear. Of note is that the calculation of the prognostic ability is principally different from the conventional calculation of a positive predictive value in that it specifically considers results preceding BOS and not results when patients have been diagnosed with BOS.

The Mann-Whitney U test was applied for tests of significant differences between the patients who developed BOS and those who did not. Standard software packages (Statistica 7; StatSoft, Inc., Tulsa, OK, USA) and Excel (Microsoft Inc., USA) software were used.

## Results

Spirometry was obtained at 470 occasions, lung volumes at 406, CO-uptake at 424, and N_2_-slope at 363 occasions. Six patients died during the follow up period. The prevalences of BOS and BOS 0-p at the various time points are shown in table 1.

**Table 1:** Number of patients divided according to the BOS stage at the regular control visits during the 3 years follow up of 61 double lung recipients.

	Months after transplantation					
	
	6	9	12	18	24	36
NoBOS	61	57	52	50	49	43

BOS 0-p	0	1	3	2	2	3

BOS 1	0	3	4	4	1	3

BOS 2	0	0	0	1	1	1

BOS 3	0	0	2	4	8	11

Table 2 presents median values of relevant pulmonary function variables obtained 6 and 36 months after transplantation. Six months after transplantation the CO-uptake was substantially reduced and the N_2_-slope abnormally increased, but there were no significant differences between NoBOS and BOS patients. Thirty-six months post transplantation the NoBOS patients remained essentially unchanged whereas among BOS patients FEV_1_, FVC, RV and N_2_-slope had deteriorated and were significantly different from the NoBOS patients.

**Table 2:** Median values of lung function results expressed in percent of the predicted normal value.

Lung function variable	**6 months post transpl**.		**36 months post transpl**.		
	
	NoBOS	BOS	NoBOS	BOS	p value*
FEV1	83	74	88	44	< 0,001

FVC	76	76	89	64	< 0,001

RV	108	93	89	132	0,011

TLC	87	78	91	89	0,505

CO uptake	52	56	61	53	0,256

N_2-_slope	189	170	211	950	< 0,001

P-values refer to differences between NoBOS and BOS 36 months post transplantation

Table 3 shows the prevalence of normal and abnormal test results and 3b the resulting operating characteristics. Thus, the sensitivity to diagnose BOS and BOS 0-p of the N_2_-slope in percent predicted normal was almost as high as for FEV_1 _< 90% of the basal value (96% versus 100%, table 4). The corresponding positive predictive values were also similar but much lower. The results regarding TLC, FRC, RV and CO-uptake are not shown as the sensitivities were very low.

**Table 3:** Number of normal and abnormal test results divided according to the current diagnostic status among 58 double lung transplant recipients.

	Dimension	Cut off value	NoBOS		BOS		BOS 0-p	
			
			Normal	Abnormal	Normal	Abnormal	Normal	Abnormal
FEV1	% basal	< 90	264	28	0	37	0	7
FVC	% basal	< 90	276	11	8	27	5	1
N_2 _slope	% basal	> 299	240	12	12	13	4	3
FEV1	% pred.	< 49	279	14	12	24	7	0
FVC	% pred.	< 52	275	13	24	10	6	0
N_2 _slope	% pred.	> 478	234	18	1	24	0	7

Cut off values are based on the 43 patients who did not develop BOS or BOS 0-p and are chosen to give 95% specificity except for FEV < 90% of the basal value.

**Table 4:** Operating characteristics of the prevalences shown in a.

	Dimension	Specificity (%)	Sensitivity (%)		Positive predictive value (%)		Negative predictive value (%)	
			
			BOS	BOS 0-p	BOS	BOS + BOS 0-p	BOS	BOS + BOS 0-p
FEV1	% basal	90	100	100	57	61	100	100
FVC	% basal	96	77	17	71	72	97	96
N_2 _slope	% basal	95	52	43	52	57	95	94
FEV1	% pred.	95	67	0	63	63	96	94
FVC	% pred.	95	29	0	43	43	92	90
N_2 _slope	% pred.	93	96	100	57	63	100	100

BOS 0-p preceded BOS in 4 of the 15 BOS patients, giving a sensitivity of 27%. There were 46 patients who did not develop BOS during the follow up period and 43 of these were not diagnosed as BOS 0-p resulting in specificity of 93%. There were three subjects diagnosed as BOS 0-p where the development of BOS was uncertain and thus the positive predictive value may be considered to be 57%.

**Figure 1:**
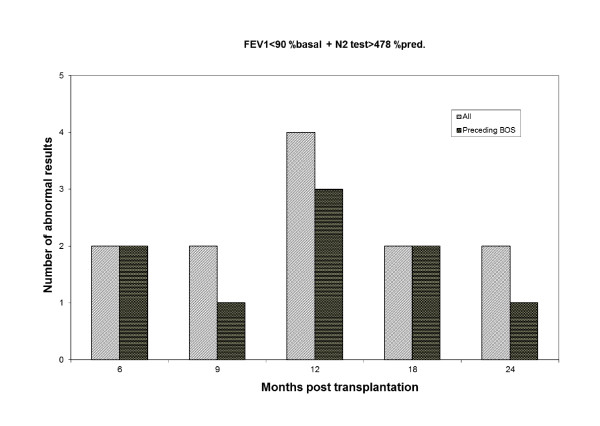
**Ratio of FEV1 < 90% basal and N2-test > 478% predicted over time**. Figure 1 illustrates the number of occasions over time (n = 12) when the test results of both FEV1 < 90% basal and N2-test > 478% predicted were abnormal. Black bars denote when abnormal values preceded BOS (n = 9).

The ability of a single test result to predict BOS was determined regarding FEV_1 _< 90% of the basal value, and regarding the N_2_-slope > 478% of the predicted normal value. Table 5 shows that if a FEV_1 _result is < 90% of the basal value, the probability of that result to precede BOS is 31% whereas the corresponding probability for the N_2_-slope > 478% of predicted normal is 56%. Furthermore, both tests were "abnormal" simultaneously at 12 instances and 9 of these preceded BOS. Consequently, there is a 75% probability that this combination predicts the development of BOS. Figure 1 illustrates the prevalence over time of both test results simultaneously being abnormal. The predictive ability does not appear to change systematically over time.

**Table 5:** Results of FEV1 and the N2 test from 6 to 24 months post transplantation.

	Definition of abnormal	Total number of test results	Total number of abnormal results	Number of abnormal results preceding BOS	Predictive value (%)
FEV1	< 90% basal	299	28	11	39

N2 test	> 478% pred.	259	22	12	55

FEV1 + N2 test	Both definitions	259	12	9	75

Results in NoBOS patients and in those preceding the diagnosis of BOS are included

## Discussion

The present study shows that an abnormal N_2_-slope in combination with a FEV_1 _result < 90% of the basal value, predicts the development of BOS with a probability of 75%. Using either FEV_1 _or the N2-slope by themselves were not as useful. The single-breath N_2_-slope identified BOS and BOS 0-p in almost all cases, but since there were abnormal values among patients without BOS and BOS 0-p the positive predictive values were lower.

The BOS 0-p stage, *i.e*. an average decline in FEV_1 _of 10-19% of the basal value of two measurements at least 3 weeks apart, was added in 2002 to the BOS scoring system in an attempt of early detection of bronchiolitis obliterans [7]. Hachem et al reported a positive predictive value of BOS 0-p of 79% based on an analysis of 183 bilateral recipients including 72 BOS 0-p patients [8]. Our results indicate that the positive predictive value of diagnosed BOS 0-p is only 57%, but this value is based on a lower patient number and on the assumption that three patients who were diagnosed as BOS 0-p at the very last control visit did not develop BOS. If only one of these three BOS 0-p patients in fact did develop BOS, the positive predictive value would have been 71%.

Measurements of ventilation distribution by single-breath washouts for N_2_, helium, and sulfur hexafluoride have been shown to be more sensitive than conventional ventilation measures in the detection of ventilation disturbances in the peripheral airways in smokers, asthma, and cystic fibrosis [16,20,21]. Especially the slope of the single-breath N_2 _test proved highly sensitive in the detection of early disturbances of ventilation in the peripheral airways caused by tobacco smoke [16]. In that study, based on 97.5% specificity, 58% of 50 years old male heavy smokers (> 15 g/d) had abnormally steep N_2 _slope whereas only 28% had abnormal FEV1. Thus, there is comprehensive amount of evidence showing that single breath wash out tests are useful in early detection of abnormalities in peripheral airways.

A recent study by Van Muylem et al [22] showed that the helium slope of the single-breath washout was more sensitive in detecting BOS stage 0-p than exhaled biomarkers, *i.e*. NO and CO. Other authors have analysed the usefulness of the single-breath N_2 _slope to diagnose BOS. Reynaud-Gaubert et al [11] used the single-breath N_2 _slope similarly as in the present study and reported a sensitivity of 100% in the detection of BOS in 47 patients after heart-lung or double lung transplantation. In that study, however, the chosen cut off value of 3% N_2_/L obtained at least twice, was acquired in 6 out of 25 patients without BOS resulting in a positive predictive value of 78.5%. Estenne et al measured the slope of the alveolar plateau of various gases following inhalation of 1 L of a gas mixture of helium and sulfur hexaflouride and also found a very high sensitivity. The associated cut off values were defined by the confidence interval obtained in 10 stable lung transplant recipients, resulting in a specificity of 82% [12].

As any sensitivity may be achieved by choosing various cut off values, it is desirable that similar specificities are applied to facilitate comparisons. Regarding lung function tests it is generally accepted that the lower limit normal is defined as to result in 95% specificity. Accordingly the cut off value in the present study was chosen so that 95% of all measurements from 6 months post transplantation onward of the 43 stable patients were defined as normal. There appears to be no doubt in that indexes of ventilation distribution are in excellent agreement with the BOS diagnosis, i e having high sensitivitiy, but that abnormal values occur to some extent also among those patients who do not have BOS, resulting in less favorable positive predictive values.

The ability of various indexes of ventilation distribution to predict BOS was indicated in an earlier report [9] and has since been confirmed by others [11,12] reporting the time delay between reaching the cut off and the diagnose of BOS. Estenne et al reported that an increased alveolar slope preceded BOS in 17 of 18 BOS patients, but this was also obtained in 7 of 39 patients who did not develop BOS within the course of the study [12]. Thus the positive predictive value was 71%. The present study shows that the probability of a single test result of the N_2 _slope to precede BOS within the three years follow up is only 56%, but when combined with concomitant FEV_1 _< 90% of the basal value, the probability increases to 75%, a value that may be considered clinically useful. A drawback is the relatively low prevalence of this combination. Considering the moderate regular control scheme in our transplantation unit compared to previous reports, the analysis based on single test results appears suitable but will necessarily result in more false positive results. Furthermore, as the number of BOS cases was rather small in the present study as well as in previous similar studies, the calculated characteristics are based on small numbers. Another limitation to our study is the retrospective study design which is a risk that the results can be more observational. Preferably, one should apply the N_2_-test in a larger patient cohort and in a prospective manner to ascertain how it would serve as an early predictor of BOS.

The data available for the study was a retrospective analysis of patients from 1990 up until 2003. The intention was to have a stable immunosuppressive protocol as well as CMV prophylaxis regimen for the studied cohort and minimize for possible confounding factors. Since 2003 the regime and prophylaxis have been altered according to new international guidelines.

The pulmonary function laboratory at our institution has considerable experience of the N_2 _test and all technicians performing the tests were experienced and used to the method. All measurements were performed blinded to the status of the patient. Furthermore, the applied algorithm for the determination of the slope is robust [16], *i.e*. the difference of N_2 _concentration between the closing point and TLC - 0,825 mL BTPS divided by the corresponding volume. The chosen cut off values depend on this algorithm and also on the applied reference equation [19] since the cut off values are expressed in percent of predicted normal.

A diagnosis of BOS depends on exclusion of other possible causes of a decline in FEV_1 _[23]. In the present material, the numerous routinely performed clinical examinations, chest radiographs and surveillance bronchoscopies with BAL and TBB ensure that possible confounding factors are reasonably controlled for. The NoBOS patients, however, defined as not having BOS at the follow-up examinations, probably contain some patients who later will develop BOS. This circumstance may certainly influence the analysis, but presumably does not influence the comparison of the N_2_-slope and BOS 0-p regarding the predictability.

The treatment of BOS has over the years been unsuccessful, but more recently several promising therapeutic approaches have been proposed [24-26]. An intensified treatment at an early stage of the disease, *i.e*. BOS 0-p might prevent the irreversible functional impairment which otherwise would occur.

## Conclusions

We conclude that a single abnormally steep N_2_-slope in combination with a concomitant FEV_1 _< 90% of the basal value, is clinically useful in predicting BOS. Follow-up protocols of heart-lung and bilateral lung transplant recipients should preferably include lung function tests sensitive to obstruction of the small peripheral airways, e g single-breath washout tests.

## Competing interests

The authors declare that they have no competing interests.

## Authors' contributions

GCR participated in the conception and design of the study, the analysis and interpretation of the data and the drafting of the manuscript. GM participated in the conception of the study, the analysis and interpretation of the data and the revision of the manuscript. BH participated in the measurements and the analysis of the data as well as the revision of the MS. BB participated in the conception, design and coordination of the study, in the measurement of the data, the analysis of the data and drafting of the MS. All authors read and approved the final manuscript.

## References

[B1] Estenne M Hertz MI Bronchiolitis obliterans after human lung transplantation Am J Respir Crit Care Med 2002 166 440 444 10.1164/rccm.200201-003PP 12186817

[B2] Trulock EP Edwards LB Taylor DO Boucek MM Keck BM Hertz MI Registry of the International Society for Heart and Lung Transplantation: twenty-third official adult lung and heart-lung transplantation report--2006 J Heart Lung Transplant 2006 25 880 892 10.1016/j.healun.2006.06.001 16890108

[B3] Fietta AM Meloni F Lung transplantation: the role of azithromycin in the management of patients with bronchiolitis obliterans syndrome Curr Med Chem 2008 15 716 723 10.2174/092986708783885228 18336286

[B4] Hachem RR Yusen RD Meyers BF Aloush AA Mohanakumar T Patterson GA Trulock EP Anti-human leukocyte antigen antibodies and preemptive antibody-directed therapy after lung transplantation J Heart Lung Transplant 2010 29 973 980 10.1016/j.healun.2010.05.006 20558084 PMC2926224

[B5] Chamberlain D Maurer J Chaparro C Idolor L Evaluation of transbronchial lung biopsy specimens in the diagnosis of bronchiolitis obliterans after lung transplantation J Heart Lung Transplant 1994 13 963 971 7865530

[B6] Cooper JD Billingham M Egan T Hertz MI Higenbottam T Lynch J Mauer J Paradis I Patterson GA Smith C A working formulation for the standardization of nomenclature and for clinical staging of chronic dysfunction in lung allografts. International Society for Heart and Lung Transplantation J Heart Lung Transplant 1993 12 713 716 8241207

[B7] Estenne M Maurer JR Boehler A Egan JJ Frost A Hertz M Mallory GB Snell GI Yousem S Bronchiolitis obliterans syndrome 2001: an update of the diagnostic criteria J Heart Lung Transplant 2002 21 297 310 10.1016/S1053-2498(02)00398-4 11897517

[B8] Hachem RR Chakinala MM Yusen RD Lynch JP Aloush AA Patterson GA Trulock EP The predictive value of bronchiolitis obliterans syndrome stage 0-p Am J Respir Crit Care Med 2004 169 468 472 14670802 10.1164/rccm.200307-1018OC

[B9] Gilljam M Thylén A Riise G Nilsson F Mårtensson G Single breath nitrogen test as a predictor of bronchiolitis obliterans in heart lung and bilateral lung transplant recipients Eur Resp J 1997 10 2341

[B10] Anthonisen NR Danson J Robertson PC Ross WR Airway closure as a function of age Respir Physiol 1969 8 58 65 10.1016/0034-5687(69)90044-9 5366416

[B11] Reynaud-Gaubert M Thomas P Badier M Cau P Giudicelli R Fuentes P Early detection of airway involvement in obliterative bronchiolitis after lung transplantation. Functional and bronchoalveolar lavage cell findings Am J Respir Crit Care Med 2000 161 1924 1929 10852768 10.1164/ajrccm.161.6.9905060

[B12] Estenne M Van Muylem A Knoop C Antoine M Detection of obliterative bronchiolitis after lung transplantation by indexes of ventilation distribution Am J Respir Crit Care Med 2000 162 1047 1051 10988128 10.1164/ajrccm.162.3.9912063

[B13] Riise GC Andersson BA Kjellström C Mårtensson G Nilsson FN Ryd W Schersten H Persistent high BAL fluid granulocyte activation marker levels as early indicators of bronchiolitis obliterans after lung transplant Eur Respir J 1999 14 1123 1130 10.1183/09031936.99.14511239 10596701

[B14] Cotes JE Chinn DJ Quanjer PH Roca J Yernault JC Standardization of the measurement of transfer factor (diffusing capacity). Report Working Party Standardization of Lung Function Tests, European Community for Steel and Coal. Official Statement of the European Respiratory Society Eur Respir J Suppl 1993 16 41 52 8499053

[B15] Quanjer PH Tammeling GJ Cotes JE Pedersen OF Peslin R Yernault JC Lung volumes and forced ventilatory flows. Report Working Party Standardization of Lung Function Tests, European Community for Steel and Coal. Official Statement of the European Respiratory Society Eur Respir J Suppl 1993 16 5 40 8499054

[B16] Oxhöj H Bake B Measurement of closing volume with the single breath nitrogen method Scand J Respir Dis 1974 55 320 331 4462206

[B17] Oxhöj H Bake B Wilhelmsen L Ability of spirometry, flow-volume curves and the nitrogen closing volume test to detect smokers. A population study Scand J Respir Dis 1977 58 80 96 857303

[B18] Salorinne Y Single-breath pulmonary diffusing capacity. Reference values and application in connective tissue diseases and in various lung diseases Scand J Respir Dis Suppl 1976 96 1 84 1076826

[B19] Sixt R Bake B Oxhöj H The single-breath N2-test and spirometry in healthy non-smoking males Eur J Respir Dis 1984 65 296 304 6723839

[B20] Van Muylem A Baran D Overall and peripheral inhomogeneity of ventilation in patients with stable cystic fibrosis Pediatr Pulmonol 2000 30 3 9 10.1002/1099-0496(200007)30:1<3::AID-PPUL2>3.0.CO;2-L 10862156

[B21] Gustafsson PM Ljungberg HK Kjellman B Peripheral airway involvement in asthma assessed by single-breath SF6 and He washout Eur Respir J 2003 21 1033 1039 10.1183/09031936.03.00049302 12797500

[B22] Van Muylem A Knoop C Estenne M Early detection of chronic pulmonary allograft dysfunction by exhaled biomarkers Am J Respir Crit Care Med 2007 175 731 736 10.1164/rccm.200609-1301OC 17234904

[B23] Boehler A Estenne M Post-transplant bronchiolitis obliterans Eur Respir J 2003 22 1007 1018 10.1183/09031936.03.00039103 14680094

[B24] Iacono AT Corcoran TE Griffith BP Grgurich WF Smith DA Zeevi A Smaldone GC McCurry KR Johnson BA Dauber JH Aerosol cyclosporin therapy in lung transplant recipients with bronchiolitis obliterans Eur Respir J 2004 23 384 390 10.1183/09031936.04.00058504 15065826

[B25] Verleden GM Vanaudenaerde BM Dupont LJ Van Raemdonck DE Azithromycin reduces airway neutrophilia and interleukin-8 in patients with bronchiolitis obliterans syndrome Am J Respir Crit Care Med 2006 174 566 570 10.1164/rccm.200601-071OC 16741151

[B26] Fischer GB Sarria EE Mattiello R Mocelin HT Castro-Rodriguez JA Post infectious bronchiolitis obliterans in children Paediatr Respir Rev 2010 11 233 239 10.1016/j.prrv.2010.07.005 21109182

